# Nutritional value assessment of umufumba: A Rwandan wild edible plant *Mondia whytei* (Hook. F)

**DOI:** 10.1002/fsn3.796

**Published:** 2018-11-05

**Authors:** Habinshuti Janvier, Théoneste Muhizi, Jean Bernard Ndayambaje, Teresa Ayuko Akenga

**Affiliations:** ^1^ Chemistry Department College of Science and Technology University of Rwanda Kigali Rwanda; ^2^ Department of Chemistry and Biochemistry University of Eldoret Eldoret Kenya

**Keywords:** β‐carotene, minerals, *Mondia whytei*, nutrients, protein, sugars, vitamin

## Abstract

This study reports findings on the macro‐ and micronutrient content of the root bark of *Mondia whytei* Skeels (Hook. F) (Asclepiadaceae), from five Rwandan different localities Northern part, and the nutritional content was found to vary locality of the plant. Vitamins were analyzed using high‐performance liquid chromatography (HPLC). The vitamins analyzed included β‐carotene (fat soluble), niacin, riboflavin, and thiamine (water soluble) whose content was found to range from 4.4 to 73.5 *μ*g/g dry matter. Atomic absorption spectrophotometry (AAS) was used to analyze calcium, iron, copper, manganese, magnesium, zinc, cadmium, and lead, whereas flame emission spectrophotometry (FES) was used for the determination of sodium and potassium. The highest mineral (mg/g of dry matter) was K (11.34–32.05), while the lowest was Pb (0.03–0.06). Mean total protein content was determined using the combustion method, and the mean percentage range was 4.7%–15.4%. The sugars (fructose, glucose, sucrose, and xylose) were analyzed by HPLC. There were significant differences (2.5–132.2 mg/g) in the free sugar contents. The results obtained in this study indicate that *Mondia whytei* Skeels (Hook. F) is rich in nutrients and can be developed for use as a food supplement since the nutrient contents are within the ranges recommended by FAO and WHO.

## 1 INTRODUCTION

Plants are known to provide essential nutrients for human nutrition, and the mainly consumed parts of the plants are the root bark, stem bark, leaves, fruits, and seeds (Mark, Michael, & Christopher, 2000; Maundu, Ngugi, & Kabuye, 1999). A number of indigenous plants have been used as supplements in the diet, even in small quantities. The plants supply the macro‐ and micronutrients such as minerals, vitamins, proteins, sugars, which are from the roots, leaves, stem bark, and tubers (Britton, 1991; Maundu et al., 1999; Recherches ethnopharmacognosiques sur les plantes utilisées en médecine traditionnelle au Burundi occidental, 1990). Several indigenous plants have been utilized in traditional sweetening and flavoring of food, beverages, and in herbal medicine (Kinghorn, 1987). *Mondia whytei* (Hook. F.) Skeels, Asclepiadaceae, is a member of the milkweed family. It is a deciduous canopy‐climbing liane, which grows in forests, bushland, and wasteland (Agnew & Shirley, 1994; Mukazayire et al., 2011). The bark is green maturing to brown and has nodes and internodes. The leaves are green, opposite, soft, and hairy entire margins lanceolate and caudex apex. *Mondia whytei Skeels* (Hook. F) is distributed in forests and humid savannahs of tropical and South Africa. In Rwanda, for instance, it is mainly found in the country's mostly dense *Gishwati, Nyungwe,* and *Akagera* forests (La médecine indigène au Ruanda et Lexique des termes médicaux français ‐ urunyarwanda Mémoire présenté à la séance du 20 décembre, 1954), while in Kenya, this weed is commonly known as “*Mukombelo*” by the Luhya community. It takes a minimum of 2 years for the roots to mature (Kinghorn, 1987; Médecine traditionnelle et plantes médicinales rwandaises, 1987; Norah, Fedha, & Edah, 1995).

*Mondia whytei Skeels* (Hook. F) is a plant that is widely used in Africa as a medicinal herb and as a food supplement. The roots are the mostly used parts of the plant and are eaten fresh or ground into powder (Mukonyi, Ndiege, & Luvanda, 2000). Despite its folkloric uses, there is no adequate information on its nutritional status. There is a need for a detailed study to verify the nutritional value of *Mondia whytei Skeels* (Hook. F), hence the objectives of this study. The nutrients surveyed include vitamins, sugars, proteins, and minerals. Vitamins are biologically active compounds that are essential for growth and health. Lack of particular vitamins in the body causes deficiency diseases. Vitamins can be classified as fat soluble and water soluble. This classification groups the vitamins according to problems associated with their chemical analysis and common physiological characteristics (Hsieh, Pirronen, & Karel, 1983; Piironen, Syraoja, Salminen, & Pekka, 1986). Deficiencies of vitamins cause diseases that are only cured with the intake of that particular vitamin and major nutritional deficiencies of riboflavin, thiamine, and niacin have been reported (Bohdal, Gibbs, & Simmons, 1968; Monacha, 1972). For instance, thiamine deficiency causes beriberi that ultimately affects the nervous system (Bohdal et al., 1968). Studies have classified minerals as being either essential or nonessential or macro‐ and microelements (Fairweather‐ Tait, 1996; Fairweather‐Tait, Faulks, Fatemi, & Moore, 1987). Consumption of essential elements in the body is necessary for important physiological mechanisms, some of which are not clear (Fairweather‐Tait et al., 1987; Mervyn, 1980). Consumption of excess essential elements has dire consequences on human health and their deficiencies. The following elements are essential to humans: potassium (K), sodium (Na), copper (Cu), iron (Fe), phosphorous (P), zinc (Zn), magnesium (Mg), molybdenum (Mo), and calcium (Ca). Whereas those elements regarded as toxic even when consumed in traces are mercury (Hg), cadmium (Cd), lead (Pb), tin (Sn), and chromium (Cr) (Fairweather‐Tait et al., 1987; Ferguson, 1990). Considering Fe, an essential element, hemoglobin contains about 75% of the human body Fe. This element, Fe, is present in muscles as myoglobin and in most cells of the body as a coenzyme, such as cytochromes. Fe is present in food as ferric hydroxides ferric protein complexes and heme protein complexes (Mervyn, 1980). For toxic minerals, Pb serves as a good example. High Pb levels in the human body affect the hemopotic system leading to anemia. In serious cases, the nervous system is affected leading to irreversible damage of the brain and the renal system. In mild form lead, affects the central nervous system manifested in hypertension and impaired motor skills (Ferguson, 1990). Carbohydrates are the most important components in many foods. They may be present as many isolated molecules or they may be physically or chemically bound to other molecules. Individual molecules can be classified according to the number of monomers, and they contain as monosaccharides, oligosaccharides, and polysaccharides (Berit, Drissa, Cecilie, Sylvi, & Michaelson, 2000; Dunmire & Otto, 1979). Nearly, all carbohydrates are converted into glucose, which is a blood sugar, and to ribose, an important constituent of nucleotides and nucleic acids. Complex carbohydrates contain other sugar derivatives such as uronic acid and amino acids found in the cell membranes and important in metabolism processes in the body. Proteins are polymers involving mainly α‐amino acids and a thousand other different molecules. In form of amino acids, the proteins perform a multiple of structural, hormonal, catalytic, and intracellular functions essential for life. These include the biosynthesis of porphyrines, purines, pyrimidines, and urea (Hamilton & May, 1985; Holme & Hazel, 1998). The low molecular weight amino acids play a vital role as hormones. Total protein required by human is dependent on age and sex. Therefore, protein deficiency in human nutrition leads to failure of biosynthetic functions and intracellular functions involved in redox reactions. In this study, the nutritional composition of the root bark of *Mondia whytei Skeels* (Hook. F) has been quantitatively and qualitatively determined with respect to locality and age of the plant. This is necessary since the root bark is locally marketed and consumed for various reasons.

## 2 MATERIALS AND METHODS

### 2.1 Sampling and sample pretreatment

The random sampling method was used for the collection of the plant materials (Miller & Miller, 1988). The root samples were collected with respect to localities and age. The full‐grown plants (above 3 years old) were collected from five different divisions of Musanze District, Rwanda. The root bark of young plants (1½ year old) was obtained from s*hingiro* sector is positioned on 1850 m above see water [1]. The roots were washed and debarked. The root bark was air‐dried in the oven at 35°C using a Memmert 854‐schwabach. The dried root bark was ground using a milling machine (Wiley mill 1029‐A) and collected using a fine mesh sieve (1 mm diameter). An analytical balance (Mettler AE 200), with a precision of ± 0.0001 g, was used to weigh the required sample weights for analysis. The samples were kept under refrigeration. The samples were prepared in triplicates for analysis of minerals, vitamins, proteins, and carbohydrates.

### 2.2 Reagents and standards

Vitamins (β‐carotene, thiamine, riboflavin, and niacin), carbohydrates (fructose, glucose, xylose, and sucrose), standards, and papain were obtained from Sigma‐Aldrich limited. Potassium hydroxide, sodium sulfite, acetic acid, and ascorbic acid were obtained from Sigma‐Aldrich chemicals, BDH Poole chemical England, and Prolabo Kenya as a collaboration.

### 2.3 Solvents

All solvents used during the analysis of the vitamins were HPLC grade solvents obtained from Sigma‐Aldrich and Prolabo Kenya Ltd.

### 2.4 Glassware

The glassware used in the analysis for vitamins was cleaned by soaking them in chromic acid followed by overnight soaking in detergent. Prior to each analysis, the glassware was rinsed with distilled de‐ionized water. Apparatus used for β‐carotene analysis was rinsed with methanol before analysis at each step. The pipettes and measuring cylinders were calibrated for accurate measurements, by delivering a volume of distilled de‐ionized water that was weighed at room temperature. This was repeated six times for each apparatus. The average volume and standard deviations were obtained. The apparatus with means that had no significant difference was used for analysis and measurements.

### 2.5 Analysis of vitamins (Bushway & Bureau, 1986; Craft, 1992; Kamman, Labuza, & Wartesen, 1980) HPLC instrument and operating conditions

An HPLC instrument Shimadzu liquid chromatograph with an LC‐10A Shimadzu pump, SPD‐10AV Shimadzu UV‐vis detector, was used. The readout for recording the peaks was a Shimadzu Chromatopac C‐R6A. An analytic column ODS II (250 × 4 mm i.d, 5 μm) was used for the analysis of β‐carotene, while a lichrosphere column ODS I (250 × 4 mm i.d, 5 μm) was used for riboflavin, niacin, and thiamine. The sample (10 μl) and the standard solutions were injected for each analysis using a Hamilton injector. The sensitivity of the detector was 0.005 absorbance units full scale (AUFS), but 0.05 AUFS was applied for the analysis of thiamine. β‐carotene was analyzed at a wavelength of 453 nm at a flow rate of 1 ml/min and a column temperature maintained at 25°C. Thiamine riboflavin and niacin were analyzed at a wavelength of 280 nm. The flow rate was 0.5 ml/min, and the column temperature maintained at 25°C. The lamp used was deuterium lamp. The chart speed was 0.2 cm/min and an operating backpressure of 85–90 psi.

### 2.6 Quantification of results and statistical analysis

**Figure 1:**
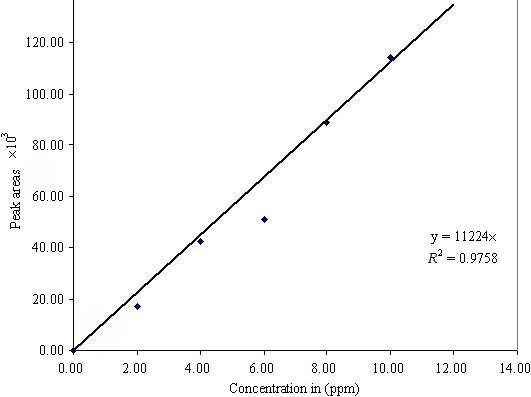
Correlation curve for riboflavin standards

The peak areas were directly proportional to the concentration in the examined range. Peak identification was achieved by comparing the retention times of the standards and the sample solutions. The standard solution was injected first to obtain the desired calibration curve followed by injections of sample extract. A linear calibration curve for each vitamin was constructed. Figure 1 is such a curve for riboflavin. Quantification was achieved by comparing the peak areas of the samples to the peak areas of the standards. The concentration data obtained for vitamins were statistically analyzed using analysis of variance (ANOVA) Turkey studentized range (HSD) test (SAS) statistical version 8.2.

### 2.7 Analysis of β‐carotene

The sample was homogenized using a mechanical blender, and the root powder (20 g) was weighed and transferred into a round‐bottomed flask (150 ml). Cold acetone (100 ml), sodium sulfite (20 g) as the desiccant, and ascorbic acid (0.3 g) as the antioxidant were added. The extract was steeped in acetone until there was no coloration. The mixture was filtered under vacuum through a Buchner funnel fitted with a Whatman filter paper (No. 42). The extract was washed four times to remove all color. The extract was evaporated almost to dryness at 35°C using a rotary evaporator. The resultant solid was treated with ethanolic KOH (5 ml, 60%), and ascorbic acid (50 μg/ml) was added as the antioxidant. The mixture was dispersed into solution and diluted with NaCl (10%) in water. The carotenoids were extracted from the sample mixture using n‐hexane: diethyl ether (70:30). The extract was evaporated almost to dryness at 35°C and the residue redissolved in n‐hexane (30 ml). An aliquot (10 μl) was injected into HPLC for analysis.

### 2.8 Analysis of niacin, riboflavin, and thiamine

**Figure 2:**
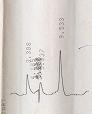
Partial high‐performance liquid chromatograms for riboflavin standard at 6 ppm

**Figure 3:**
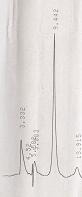
Partial high‐performance liquid chromatograms for riboflavin standard at 10 ppm

**Figure 4:**
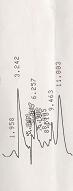
Partial high‐performance liquid chromatograms for riboflavin in root extract of *M. whytei*

Isocratic, a reversed phase eluant of the system of water: acetonitrile: acetic acid (40:59.5:0.5), was used for analysis of the above three vitamins. The sample was homogenized using a mechanical blender. The homogenate sample (10 g) was accurately weighed and transferred into a round‐bottomed flask (100 ml). HCl (20 ml, 0.26 M) was added to each sample and the mixture stirred. The mixture was hydrolyzed over boiling water for 1 hr. The pH of the mixture was adjusted to 4.5 using sodium acetate (2.5 M) and then cooled to room temperature. The sample extract was incubated for 2.5 hr in the oven (45°C). The extract was cooled to room temperature and filtered under vacuum using the Whatman filter paper (No 42). The residue was centrifuged (15 min, 55 revs/min). The supernatant was transferred to a volumetric flask (50 ml) and made to the mark with distilled de‐ionized water. The mixture was filtered through the Millipore filters, and an aliquot (10 μl) was injected into HPLC for analysis. Figures 2, 3, 4 are partial chromatograms obtained during the analysis for riboflavin standards and in the root powder of *Mondia whytei Skeels* (Hook. F), at varying concentrations.

### 2.9 Determination of mineral content

The stock solutions for various minerals were prepared by taking respective weights of the salts and dissolving in minimum hydrochloric acid. The resultant mixture was placed in a volumetric flask (1,000 ml) and made to the mark with distilled de‐ionized water to make the stock solution (1,000 ppm). An atomic absorption spectrophotometer (AAS, Shimadzu –AA‐630‐12) and a flame emission spectrophotometer (FES, PTF 7, Jenways), and flame photometer were used in this analysis. Ca, Mg, Zn, Cd, Pb, Cu, Mn, and Fe were analyzed using AAS. Samples for calcium and magnesium and the working standards for their analysis were prepared by adding 1 ml of 5% lanthanum solution (Fairweather‐ Tait, 1996; Fairweather‐Tait et al., 1987; Ramos, Harnadez, & Gonzalez, 1994).

Flame emission spectrophotometer used butane/air fuel and was fitted with filters for sodium and potassium for their analysis. Wet digestions based on dry weight procedures were used during this study.

### 2.10 Determination of Ca, Mg, Na, K, Mn, and Zn content

The root powder (0.3 g) was weighed and transferred into round‐bottomed digestion tubes. The digestion mixture (3.3 ml) was made by mixing salicylic acid (6 g) with water and concentrated sulfuric acid. The sample mixture was heated using the Kjeldahl digestion block apparatus (2 hr at 180°C). The temperature was gradually increased to 280°C. During the digestion, hydrogen peroxide (2–5 drops) was continually added until the digest was clear. The mixture was then cooled to room temperature, and distilled de‐ionized (10 ml) water was added to dissolve all the digest mixture in the digestion tube. The digested sample was filtered using an ashless Whatman filter paper (No 42), transferred into a volumetric flask (50 ml), and made to the mark with distilled de‐ionized water. A blank digest was prepared in the same way as the sample digest.

### 2.11 Determination of Cd, Pb, Fe, and Cu content

The homogenate sample (0.3 g) was transferred into a round‐bottomed digestion tube. The digestion mixture (3.3 ml) was prepared by mixing concentrated nitric acid (400 ml) with concentrated perchloric acid (40 ml), and concentrated nitric acid (10 ml) was added and the mixture swirled. The mixture was allowed to stand for 2.5 hrs after which it was moderately warmed on the Kjeldahl digestion block (1 hr) in a fume cupboard until all nitric acid distilled off. The temperature was gradually increased to 250°C and heating continued for 1 hr. The mixture was cooled, and distilled water (10 ml) and sodium nitrite (2 ml) solution were added. The mixture was boiled (10 min) and the contents transferred to a volumetric flask (50 ml), and the digest was made to the mark with distilled de‐ionized water. A blank digest was prepared in the same way as the sample digest. The standards were aspirated to give a linear relationship at different concentrations. The distilled de‐ionized water was aspirated to zero the instrument.

### 2.12 Analysis of total protein

Total protein analysis for the root samples was carried out using the combustion method (Conancher, 1990). An autosensitive nitrogen carbon analyzer Sumigraph NC‐9A (940830 series) coupled with a gas chromatograph (GC‐8A) with a thermal conductivity detector and chromatopac—6A recorder was used. The analysis range for nitrogen was 0.005%–100%, analysis time of 4–5 min/cycle. The reaction furnace temperature was 850–950°C, and the reduction furnace temperature was 534°C. The homogenized sample (10 mg) was weighed and placed on the sample boat and set on the boat‐retaining rod. The sample was introduced into the reaction tube fitted with copper fillings and was simultaneously introduced into the high temperature section of the reaction tube. Acetanilide standard was analyzed after every 10 samples for stabilization of the combustion equipment and the standard peak.

### 2.13 Analysis of sugars

An HPLC instrument Shimadzu model 400 fitted with a pump (LC‐10AVP), degasser unit (DGU‐10AU), column oven (CTO‐10AVP Shimadzu), system controller (SCL‐10AVP Shimadzu), and a refractive index detector (RID‐6A Shimadzu) was used. A shodex Asahipak (NH_2_ P‐50E Shimadzu), polyamine‐bonded polymeric gel; column (250 × 4.6 mm i.d, 5 μm), was used. The operating range was 4 × 10^−6^ RIU (refractive index units), at a flow rate of 0.5 and 0.4 ml/min. Back pressures of 126 kg/f were maintained. The column temperatures were maintained at 35°C. The homogenized sample (20 g) was weighed and transferred into a round‐bottomed flask (100 ml). Chloroform (30 ml) was added and the mixture fitted into a Soxhlet apparatus for 2 hr. The defatted sample mixture was air‐dried and transferred into a round‐bottomed flask. Ethanol (70 ml, 80%) was added, and the sample was vortexed and gently boiled at 80°C in a water bath for 3 hr. The sample mixture was filtered using a Whatman filter paper (No 42). The sample was washed 3–4 times, for exhaustive extraction. Lead acetate was added to the sample mixture which was further filtered and the filtrate centrifuged (15 min, 55 rev/min). The mixture was decanted and the supernatant concentrated in a rotary evaporator at 35°C almost to dryness. The sample was dissolved in water and made to 15 ml. The standard and the sample (10 μl of each) were injected for each analysis (Bowers, 1991; Carrel, Brilllouet, & Thibault, 1985; Fellman, Artz, Tassinari, Cole, & Augustin, 1992).

## 3 RESULTS AND DISCUSSION

### 3.1 Vitamin content in root bark of *M. whytei*

In the present study, fat‐soluble and the water‐soluble vitamins were assayed. The equipment used was simple and gave reproducible and accurate results.

### 3.2 Fat‐soluble β‐carotene content

The procedure of analysis used was alkaline hydrolysis followed by solvent extraction to remove unsaponifiable materials. Alkaline hydrolysis as a digestive method helped to break down the plant material and liberate the vitamins from the protective coatings. Apart from converting the vitamin esters to their corresponding alcohols, alkaline hydrolysis also converts fats to soaps, which can be separated from vitamins by solvent extraction. Similarly, a large number of pigments and other substances that might interfere with the instrument are broken down into small water‐soluble molecules. The analyzed samples showed the presence of β‐carotene and the concentration varied with respect to age and localities. The mean concentrations for the full‐grown samples are given in Table 1 and were significantly different at the 95% confidence level. The trend showed variations for samples collected from different localities and sample harvested at different ages, hence ecological and age influence on the contents of β‐carotene in the plant. β‐Carotene concentrations ranged from a low value of 4.4 ± 0.1 μg/g (from Kinigi) to the highest value of 21.8 ± 1.7 μg/g (from Kinkware) (Table 1). The Byangabo young sample had higher concentrations (10.1 ± 0.8 μg/g) compared to the old sample (8.0 ± 0.5 μg/g). The concentrations obtained for samples collected from different localities were all above 4 μg/g. From these results, the old sample from Kinkware would be the best source of β‐carotene.

**Table 1:** β‐carotene content in *M. whytei* root bark

Locality	Age at harvest	Conc. (μg/g) ± Standard error (N = 3)
Mukinga	Old	17.8 ± 0.5 a
Busogo's	Old	11.6 ± 0.9b
Kinkware	Old	21.8 ± 1.7a
Byangabo	Old	8.0 ± 0.5 b, c
Byangabo	Young	10.1 ± 0.8 b
Kinkware	Old	4.4 ± 0.1 c

Means with the same letters in a column are not significantly different (*p* < 0.05) by Turkey's studentized range (HSD) test.

### 3.3 Water‐soluble—niacin, riboflavin, and thiamine—contents

In this study, an acid solution of 0.26 M hydrochloric acid was used and the pH adjustment to 4.5 was done. This extraction technique resulted in greater vitamin concentration. The use of UV absorbance for detection enabled the detection of thiamine without conversion to thiochrome and also permitted simultaneous measurement with niacin and riboflavin. Thiamine is strongly ionic, while riboflavin and niacin are nonionic.

The mobile phase used gave reproducible results for the peak areas obtained for the three vitamins and allowed the simultaneous determination of thiamine, riboflavin, and niacin. Statistical correlation coefficients obtained were 0.998325 for niacin, 0.97377 for riboflavin, and 0.97983 for thiamine. The detection limit for vitamins was 1.0, 0.5, and 0.5 μg/L for thiamine, riboflavin, and niacin, respectively. Table 2 gives the concentrations of water‐soluble vitamins obtained from the root bark of *Mondia whytei Skeels* (Hook. F). The niacin content showed varied differences. The highest concentration was for a sample collected from Kinigi (81.6 ± 1.6 μg/g), and the lowest was from Mukinga (5.3 ± 0.3 μg/g). The young Byangabo sample contained significantly high concentrations (73.5 ± 1.8 μg/g) of niacin. The concentration ranges of riboflavin were between 24.5 ± 1.3 μg/g from Busogo's and 6.5 μg/g from Byangabo, while those of thiamine were 37.0 ± 2.1 μg/g from Kinkware and 7.8 ± 0.2 μg/g from Byangabo. Notably, the concentration of riboflavin for the young sample (18.5 ± 0.4 μg/g) was about three times higher than that of full‐grown sample from Byangabo. The niacin content was, in most cases, relatively higher than those of other water‐soluble vitamins, with exception of the sample from Mukinga. Variations in contents of these water‐soluble vitamins show the ecological effects on vitamin contents. Generally, the lowest concentrations were recorded from the samples collected from Byangabo for thiamine and riboflavin, while Mukinga had the lowest concentration for niacin. The values obtained in this study compare well with those reported for other plants. For example, riboflavin content of green grams (5.0 μg/g), beans (2.6 μg/g), carrots (56.8 μg/g), and Irish potatoes (20.0 μg/g) has been reported (Nyambaka, 1988). The concentrations were also within the required daily intakes of these vitamins by man (F.A.O., 1988). Hence, the root sample of this plant can be used as a supplement for the water‐soluble vitamins.

**Table 2:** Water‐soluble vitamins content *M. whytei* root bark

Localities	Age	Concentrations μg/g ±Standard error (N = 3)		
		Niacin	Riboflavin	Thiamine
Mukinga	Old	5.3 ± 0.3d	15.5 ± 0.3c	10.7 ± 0.2c
Busogo's	Old	32.8 ± 0.8c,d	24.5 ± 1.3a	13.4 ± 0.8c
Kinkware	Old	62.9 ± 1.7b,c	14.1 ± 0.3c	37.0 ± 2.1a
Byangabo	Old	14.4 ± 1.1d	6.5 ± 0.0d	7.8 ± 0.2 c
Byangabo	Young	73.5 ± 1.8a	18.5 ± 0.4b	40.1 ± 1.0a
Kinkware	Old	81.6 ± 1.6b	15.8 ± 0.6b,c	21.4 ± 0.9b

Means with the same letters in a column are not significantly different (*p* < 0.05) by Turkey's studentized range (HSD) test.

### 3.4 Mineral content in the root bark powder of *Mondia whytei Skeels* (Hook. F)

The mean concentrations of minerals for the old and young root bark are listed in Tables 3 and 4. The discussion below has grouped the minerals according to their chemical classifications.

**Table 3:** Mineral content *M. whytei* root bark

Minerals	Concentrations mg/g (± standard error)				
	Mukinga	Busogo's	Kinkware	Byangabo	Kinkware
Potassium	20.45 ± 0.00b	16.31 ± 0.48c	11.34 ± 0.48e	13.83 ± 0.00d	32.05 ± 0.00a
Sodium	15.71 ± 0.00b	7.30 ± 0.00d	5.61 ± 0.00d	10.66 ± 0.97c	24.13 ± 0.00a
Magnesium	2.84 ± 0.01a	2.07 ± 0.00b	2.93 ± 0.06a	1.40 ± 0.01c	1.99 ± 0.03b
Calcium	8.26 ± 0.02a	4.53 ± 0.07c	6.90 ± 0.04b	3.09 ± 0.16d	4.51 ± 0.07c
Iron	0.34 ± 0.03a,b	0.30 ± 0.01b,c	0.21 ± 0.01c	0.22 ± 0.00b,c	0.44 ± 0.01a
Zinc	0.05 ± 0.00b	0.05 ± 0.00b	0.07 ± 0.00a	0.04 ± 0.00b	0.05 ± 0.00b
Copper	0.00(3)± 0.00b	0.00(4)±0.00b	0.01 ± 0.00a	0.01 ± 0.00a	0.00(4)±0.00b
Manganese	0.05 ± 0.00a	0.05 ± 0.00a	0.05 ± 0.00a	0.04 ± 0.00b	0.04 ± 0.00b
Lead	0.05 ± 0.00a,b	0.03 ± 0.00b	0.06 ± 0.00a	0.03 ± 0.00b	0.06 ± 0.01a
Cadmium	0.09 ± 0.02a	0.05 ± 0.01a	0.05 ± 0.02a	0.09 ± 0.00a	0.08 ± 0.01a

Means with the same letters in a column are not significantly different (*p* < 0.05) by Turkey's studentized range (HSD) test.

**Table 4:** Mineral content *M. whytei* root bark harvested at different ages

Mineral	Concentrations in mg/g (± standard error)	
	Byangabo Old (3 years)	Byangabo Young(1.5 years)
Potassium	13.83 ± 0.00 a	5.54 ± 0.00c
Sodium	10.66 ± 0.97b	2.25 ± 0.10d,e, f
Magnesium	1.40 ± 0.00e, f, g	3.77 ± 0.02d
Calcium	3.09 ± 0.16d	2.65 ± 0.09d,e
Iron	0.22 ± 0.00 g	0.84 ± 0.03f,g
Zinc	0.04 ± 0.00 g	0.07 ± 0.01 g
Copper	0.01 ± 0 .00 g	0.01 ± 0.00 g
Manganese	0.04 ± 0.00 g	0.11 ± 0.00 g
Lead	0.03 ± 0.00 g	0.06 ± 0.00 g
Cadmium	0.09 ± 0.00 g	0.05 ± 0.00 g

Means with the same letters in a column are not significantly different (*p* < 0.05) by Turkey's studentized range (HSD) test.

#### 3.4.1 Sodium (Na) and potassium (K) content

The concentration of Na in the sample varied considerably with respect to locality (Table 3). The sample from Kinigi had the highest concentration of 24.13 mg/g, and the lowest was from Kinkware (5.61 ± 0.48 mg/g). The full‐grown Byangabo root sample contained 10.66 ± 0.97 mg/g compared to the young plant, which had 2.25 ± 0.1 mg/g (Table 4). While K content was highest in the sample from Kinigi (32.05 ± 0.00 mg/g) and lowest in the sample from Kinkware (11.34 ± 0.48 mg/g). Variation in K concentration was noted for the sample harvested at different ages (Table 4). The root bark sample of the full‐grown sample had the highest concentration of 13.83 mg/g while the young plant contained (5.54 mg/g). The concentrations of Na and K obtained were within the range of concentration of these minerals reported in plant tissues (Isaacs, 1980), and *Mondia whytei Skeels* (Hook. F) root bark can be used as a nutritional supplement.

#### 3.4.2 Calcium (Ca) and Magnesium (Mg) content

The concentration of Ca was highest in the plant samples from Mukinga (8.26 ± 0.02 mg/g) and lowest for a sample from Byangabo (3.09 ± 0.16 mg/g) (Table 3). The concentrations of the samples from different localities were significantly different at 95% confidence limits, but no significant variations with age. The mature plant had a concentration of 3.09 ± 0.16 mg/g, while the young plant contained 2.65 ± 0.09 mg/g of Ca. Magnesium content was high in the plant samples from Kinkware (2.93 mg/g) and the lowest for Byangabo. The young plant sample from Byangabo had the highest concentration of (3.77 mg/g) compared to that of the old plant (1.40 mg/g; Table 4).

#### 3.4.3 Iron (Fe) and Zinc (Zn) content

The Fe content was high for samples from Kinigi (0.44 mg/g) and the lowest for Kinkware (0.21 mg/g). The young plant contained 0.84 mg/g of Fe compared to a full‐grown plant (0.22 mg/g). The values obtained for Fe content compare well with those obtained by Ouma (1994) where the root barks had greater than 0.3 mg/g Fe content. The Zn content was highest for plant samples from Kinkware (0.07 mg/g) and lowest for those from Byangabo (0.04 mg/g). The concentration for the young plant (0.07 mg/g) was almost twice that of the full‐grown plant (0.04 mg/g) (Table 4).

#### 3.4.4 Manganese (Mn) and Copper (Cu)

Mn content showed slight variation with respect to locality and considerable variations with respect to age of roots at harvest. The samples from Busogo's, Kinkware, Mukinga had the highest concentration of 0.05 mg/g, and the lowest was from Byangabo and Kinigi samples (0.04 mg/g; Table 3). The young plant samples had a high concentration of 0.11 mg/g compared to that of the full‐grown sample (0.04 mg/g; Table 4). The concentration of Cu was relatively lower, compared to Mn concentration in the plant samples from all localities (Table 3). The sample from Byangabo and Kinkware had the highest concentration of 0.01 mg/g and low concentrations of 0.003 mg/g for Mukinga, with Kinigi and Busogo's (0.004 mg/g). The young sample contained (0.01 mg/g) while the mature sample contained (0.01 mg/g, Table 4).

#### 3.4.5 Cadmium (Cd) and Lead (Pb)

Levels of Cd in the root samples from different localities were not significantly different (Table 3). The concentrations were (0.09 mg/g) for samples from Byangabo and Mukinga, while samples from Busogo's and Kinkware had contents of 0.05 mg/g. The young sample contained 0.05 mg/g of Cd, while the mature sample contained (0.09 mg/g; Table 4). The levels of cadmium recorded were below the normal concentration ranges for the element in plant tissues and the recommended tolerable intakes for cadmium. The concentrations of the samples were far below the toxic levels. Lead content in the root samples for the plant from different localities was relatively low and varied considerably (Table 3). The concentrations were (0.06 mg/g) for plants from Kinkware and Kinigi, Byangabo (0.03 mg/g) (Table 4). The young plant contained 0.06 mg/g, while the mature plant contained 0.03 mg/g. The values were within the required minimum tolerable intakes of 20–514 μg/day recommended by World Health Organization (WHO) (1984). The concentrations of the elements analyzed were within the range of the concentrations found in plant tissues (Isaacs, 1980).

### 3.5 Protein content in *Mondia whytei Skeels* (Hook. F)

The protein concentrations were significantly different at 95% confidence limits (Table 5). The total protein percentage ranged from 13.4 ± 1.0% for samples from Mukinga to 4.6 ± 1.0% for a sample from Kinkware. The young plant from Byangabo showed high protein percentages of 15.4 ± 0.4%, approximately twice the percentage protein found in the mature plant (Table 5). Thus, the young plant from Byangabo and old plant from Mukinga would be preferred for the provision of crude protein.

**Table 5:** Protein content *M. whytei* root bark

Locality	Age	% Protein content (± standard error)
Mukinga	Old	13.4 ± 1.0a,b
Busogo's	Old	5.9 ± 0.2c,d
Kinkware	Old	4.6 ± 0.1d
Byangabo	Old	6.9 ± 0.1c
Byangabo	Young	15.4 ± 0.4a
Kinkware	Old	11.7 ± 0.1b

Means with the same letters in a column are not significantly different (*p* < 0.05) by Turkey's studentized range (HSD) test.

### 3.6 Sugar content in *Mondia whytei Skeels* (Hook. F)

There were significant differences in the free sugar contents of the root sample from different localities (Table 6). The concentration of fructose for the full‐grown pant was high in samples collected from Byangabo (15.3 mg/g) and the lowest for those from Mukinga (7.9 mg/g). The concentration for the sample harvested at a young age from Mlava was 42.3 mg/g compared to that of the full‐grown sample (15.3 mg/g) harvested from the same locality. The glucose content in mature samples sampled from different localities also varied considerably (Table 6). The highest concentration of glucose was found in samples from Byangabo (9.0 mg/g), while the lowest concentration was from Mukinga (2.5 mg/g). The young sample had the highest concentration of glucose (12.7 mg/g) compared to the full‐grown sample (9.0 mg/g). The concentrations of other sugars showed similar variations (Table 6). However, the samples from Byangabo predominantly gave the highest concentrations of sugars compared to samples from other localities except, for sucrose, which was high for Busogo's sample. Hence, the concentrations for sugars varied considerably with respect to age and localities.

**Table 6:** Sugar content *M. whytei* root bark

Locality	Age	Concentrations mg/g (±standard error)			
		Fructose	Glucose	Sucrose	Xylose
Mukinga	Old	7.9 ± 0.4c	2.5 ± 0.3e	22.1 ± 0.2d	9.2 ± 0.0c
Busogo's	Old	8.4 ± 1.4c	6.3 ± 0.1c	46.9 ± 2.1b	10.3 ± 1.7c
Kinkware	Old	10.4 ± 0.6c	5.6 ± 0.0c	22.5 ± 2.5d	12.8 ± 0.7c
Byangabo	Old	15.3 ± 0.6b	9.0 ± 0.04b	36.6 ± 1.6c	18.7 ± 0.7b
Byangabo	Young	42.3 ± 0.3a	12.7 ± 0.2a	132.2 ± 0.8a	51.8 ± 0.3a
Kinkware	Old	8.6 ± 0.7c	4.2 ± 0.1d	14.1 ± 0.1d	11.5 ± 0.1c

Means with the same letters in a column are not significantly different (*p* < 0.05) by

Turkey's studentized range (HSD) test.

## 4 CONCLUSION

The results obtained indicate that *Mondia whytei Skeels* (Hook. F) contained vitamins analyzed for and in substantial quantities. The samples collected from Kinkware and Kinigi showed high values of both the water and fat‐soluble vitamins analyzed. The young sample from Byangabo had a high concentration of the vitamins compared to the full‐grown sample from the same locality. The results also show that *Mondia whytei Skeels* (Hook. F) root bark from Kinkware, Kinigi, Byangabo, Mukinga, and Busogo's contains minerals, proteins, and sugars in acceptable concentrations. It is recommended that this plant is developed further into a nutritional supplement for the local and international communities.

## CONFLICT OF INTEREST

The authors declare that they do not have any conflict of interest.

## ETHICAL STATEMENTS

This study does not involve any human or animal testing.
